# Differences between antioxidant defense parameters and specific trace element concentrations in healthy, benign, and malignant brain tissues

**DOI:** 10.1038/s41598-021-94302-5

**Published:** 2021-07-20

**Authors:** Slavica Borković-Mitić, Aleksandar Stojsavljević, Ljiljana Vujotić, Siniša Matić, Bojan Mitić, Dragan Manojlović, Slađan Pavlović

**Affiliations:** 1 Department of Physiology, Institute for Biological Research, “Siniša Stanković” - National Institute of the Republic of Serbia, University of Belgrade, Bulevar despota Stefana 142, 11060 Belgrade, Serbia; 2 Faculty of Chemistry, University of Belgrade, Studentski trg 12-16, 11100 Belgrade, Serbia; 3 Innovation Center of the Faculty of Chemistry, University of Belgrade, Studentski trg, 12-16, 11000 Belgrade, Serbia; 4 Faculty of Medicine, University of Belgrade, Doktora Subotića 8, 11000 Belgrade, Serbia; 5 Clinical Center of Serbia, Neurosurgery Division, Doktora Koste Todorovića 4, 11000 Belgrade, Serbia; 6 Institute of Zoology, Faculty of Biology, University of Belgrade, Studentski trg 16, 11000 Belgrade, Serbia; 7 South Ural State University, Lenin prospect 76, Chelyabinsk, Russia

**Keywords:** Chemical biology, Physiology, Molecular medicine

## Abstract

There are only a few reports examining the impact of oxidative stress in patients with benign and malignant brain tumors. In this study we investigated whether there are changes in antioxidant system (AOS) parameters and key trace elements between control, benign and malignant brain tissues. The study also aimed to examine correlations between the analyzed parameters. The study enrolled both types of brain tumors, benign tumors (BT) and malignant tumors (MT). The results were compared with control tissue (CT) without tumor infiltration collected from patients with BT. The following antioxidant parameters were determined: activities of total, manganese-containing, and copper/zinc-containing superoxide dismutase (TotSOD, MnSOD and CuZnSOD), activities of catalase, glutathione peroxidase, glutathione S-transferase, glutathione reductase and acetylcholine esterase (AChE), the concentrations of glutathione and sulfhydryl groups and of manganese (Mn), copper (Cu), zinc (Zn), and selenium (Se). BT and MT had altered activities/levels of multiple AOS parameters as compared to CT, indicating that tumor cells had an altered cell metabolism and changes in AOS represent adaptive response to increased oxidative stress. Low MnSOD and AChE and high GST activities were significant for distinguishing between MT and CT. Malignant tissue was also characterized by lower Mn and Cu concentrations relative to CT and BT. Principal Component Analysis clearly discriminated BT from CT and MT (PC1, 66.97%), while PC2 clearly discriminated CT from BT and MT (33.03%). Most correlative relationships were associated with Se in the BT group and Cu in the MT group. The results of this study reveal differences between the AOS parameters and the essential trace elements between the analyzed groups. The observed dysregulations show that oxidative stress could have an important role in disrupting brain homeostasis and its presence in the pathogenesis of benign and malignant brain tumors.

## Introduction

Oxidative stress is the devastating result of unregulated production of reactive oxygen species (ROS), such as hydrogen peroxide, superoxide, and the highly reactive hydroxyl radicals, as well as reactive nitrogen species (RNS). Oxidative stress can be defined as the outcome of the imbalance between the production of ROS and RNS, and the efficiency of enzymatic and non-enzymatic antioxidant defense^1^. Redox abnormalities favoring oxidative reactions cause oxidative damage of bio (macro) molecules. The products of oxidative modification disrupt cell metabolism and signaling by increasing the production of pro-inflammatory cytokines, modifying gene expression, and promoting apoptotic or necrotic cell death^1^. ROS can be generated in excess from endogenous sources (mitochondria and peroxisomes, but also from the activation of inflammatory cells or the oxidation of neurotransmitters), as well as from exogenous sources, such as different agents present in the environment, radiation, drugs, and chemicals^2^. High oxygen consumption, relatively low antioxidant levels and low regenerative capacity lead to damage of brain tissue because of oxidative stress^3^. The brain comprises 2% of the whole body but utilizes 20% of the oxygen consumed by the body, indicating that the brain can be the source of many more free radicals than other body tissues^4^.

Increased oxidative stress has been observed as a common feature of many types of cancer. ROS have multiple functions in tumor cells depending on the type of radical, their concentrations and intracellular localization. Cancer cells have higher metabolic levels than normal cells and their high proliferation rate is accompanied by the continuous production of increased ROS concentrations^5,6^.

Trace elements are important bioactive components in the brain physiology. The system of brain barriers, the blood–brain and blood cerebrospinal fluid barriers, play a particularly important role in the homeostasis of trace elements in the brain. Oxidative damage associated with trace elements has long been implicated in the process of cancerogenesis, as well as in the degree of malignant transformation of most types of tumors^7^. The cations of copper (Cu), manganese (Mn), and anions of selenium (Se) have unpaired electrons that allow their participation in redox processes. In fact, several biological effects of these elements can be explained by their ability to catalyze the initiation of free radical reactions (Fenton’s reaction) or the decomposition of peroxides and other unstable molecules, allowing the propagation of deleterious reactions involving free radicals^8^.

Homeostasis of trace elements is essential for the healthy development and functioning of the brain. Zinc (Zn), Mn, Cu and Se are necessary components of certain enzymes responsible for various metabolic processes in different tissues, including the brain. They are cofactors of antioxidant enzymes, such as superoxide dismutase and glutathione peroxidase, which provide protection against ROS damage. Through their actions within enzymes or in their free state, they block the destructive alteration of lipids, proteins, and/or nucleic acids by oxygen-derived free radicals, ionizing radiation, certain heavy metals, and other toxic substances^9^. Homeostasis of metals within tight physiological limits is maintained through the mechanisms of uptake, distribution, accumulation, and secretion. Each breakdown has deleterious effects on metal-regulated metabolic pathways and can be a crucial step in the pathogenesis of various diseases^10^.

Redox conditions in tumors can be assessed by determining the AOS profile of tumor tissue, as well as by measuring the activity of AOS parameters in normal/healthy tissue affected by the tumor. There are studies that have shown the non-physiological activity of specific AOS components in tumor samples from cancer patients^11,12^. However, data on brain tumors and comparative analyzes of malignant and benign tumors are still scarce, fragmentary, and inconclusive. Examination and comparative analysis of redox conditions in malignant and benign tumor tissues, along with normal tissues, could be particularly important for understanding the role of ROS in the pathophysiology of cancers.

The activities of the total, manganese and copper zinc containing superoxide dismutases (Tot SOD, Mn SOD, CuZn SOD, EC 1.15.1.1, respectively), catalase (CAT, EC 1.11.1.6), glutathione peroxidase (GSH-Px, EC 1.11.1.9), glutathione S-transferase (GST, 2.5.1.18), glutathione reductase (GR, EC 1.6.4.2) and acetylcholinesterase (AChE, 3.1.1.7), as well as the concentrations of reduced glutathione (GSH) and sulfhydryl groups (SH) were determined. The four most important essential trace elements for brain homeostasis (Mn, Cu, Zn and Se) were also determined. The main goal of our study was to determine coordination between components of the AOS and between the essential trace elements, to better understand the functioning of benign and malignant tumor tumors.

## Results

### Antioxidant defense system in brain tissues

The activities of antioxidant enzymes and AChE and the concentrations of GSH and SH are presented in Table 1, with the statistics provided in Supplementary Table S1 (one-way ANOVA of the comparison between different investigated tissue). The obtained results show that the activities of TotSOD and MnSOD were significantly lower in the BT group in comparison to the CT group (*P* = 0.028 and *P* = 0.047, respectively), while the activity of TotSOD was higher in the MT group as compared to the BT group (*P* = 0.038). GST activity was significantly higher in MT as compared to CT (*P* = 0.016) and BT (*P* = 0.048). AChE had the highest activity in the BT group, but this was only significantly different when compared to MT (*P* = 0.017). Similarly, the concentration of the SH group had the highest value in the BT group relative to both CT (*P* = 0.029) and MT (*P* = 0.022).

**Table 1:** The activities of total, Mn- and CuZn-containing superoxide dismutase (TotSOD, MnSOD, CuZnSOD), catalase (CAT), glutathione peroxidase (GSH-Px), glutathione S-transferase (GST), glutathione reductase (GR), and acetylcholinesterase (AChE), and the concentrations of glutathione (GSH) and sulfhydryl groups (SH) in control brain tissue (CT), benign tissue (BT) and malignant tissue (MT).

	Control tissue (CT)	Benign tissue (BT)	Malignant tissue (MT)	CT *vs* BT	CT *vs* MT	BT *vs* MT
TotSOD	13.5 ± 1.49	9.27 ± 1.62	11.6 ± 1.03	0.028*	0.580	0.039*
MnSOD	2.54 ± 0.14	2.14 ± 0.24	2.09 ± 0.09	0.047*	0.009*	0.963
CuZnSOD	10.9 ± 1.49	8.26 ± 1.61	9.48 ± 1.00	0.221	0.910	0.187
CAT	13.4 ± 1.28	15.3 ± 1.60	11.7 ± 1.58	0.435	0.603	0.177
GSH-Px	17.0 ± 1.98	17.7 ± 3.14	22.8 ± 4.65	0.923	0.070	0.145
GST	142 ± 17.79	126 ± 15.87	186 ± 19.80	0.589	0.016*	0.049*
GR	18.1 ± 2.47	15.5 ± 1.86	19.7 ± 2.14	0.730	0.343	0.224
AChE	25.6 ± 6.03	37.8 ± 6.96	17.6 ± 1.28	0.836	0.196	0.018*
GSH	42.3 ± 5.38	50.7 ± 7.61	45.9 ± 3.86	0.558	0.942	0.539
SH	393 ± 68.58	561 ± 86.14	364 ± 34.92	0.030*	0.794	0.022*

The data are expressed as the mean ± SE. One-way ANOVA and post hoc Fisher’s LSD test for equal N, with *P* < 0.05 as the level of significance were performed. Significant differences between groups are marked in bold and with an asterisk (**CT *vs* BT; CT **vs** MT; BT *vs* MT**).

**Figure 1:**
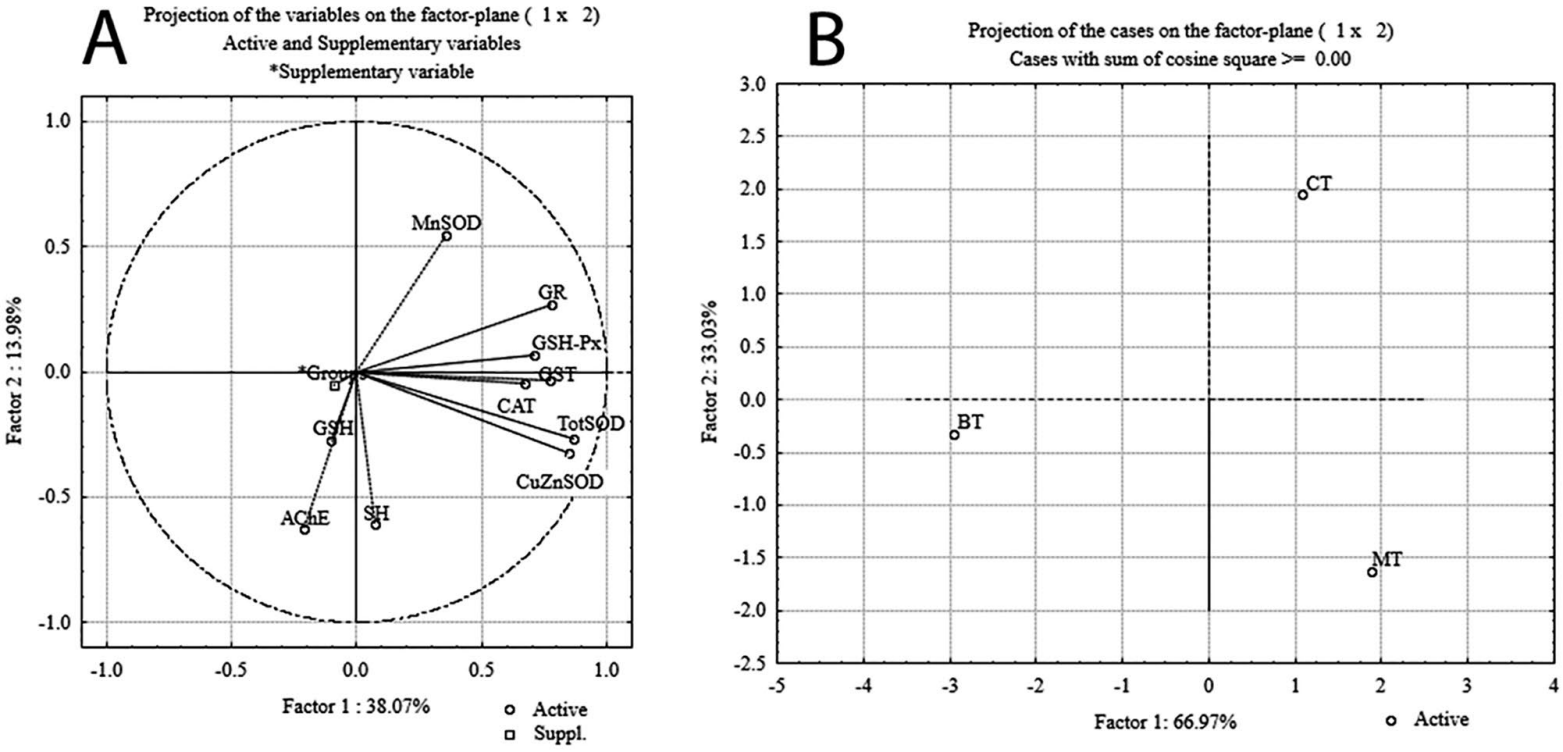
Principal Component Analysis (PCA): (**A**)-projection of the relative contribution of every antioxidant component; (**B**)-projection of groups based on antioxidant defense parameters.

Principal component analysis (PCA) was employed to examine possible discrimination of all three examined groups of tissues based on all investigated antioxidant parameters. PCA was performed in two ways as follows: by projection of the relative contribution of every antioxidant component in the factor plane (Fig. 1A), and by projection of the investigated tissues based on the antioxidant defense parameters (Fig. 1B). Table 2 shows loadings of variables onto the principal components (PCs). The PCA of the relative contribution of every antioxidant component showed that PC1 and PC2 can explain about 52% of the total variance in the data matrix. PC1 explains 38.07% of the total variance, with TotSOD, CuZnSOD and GST as the parameters that contributed most to the separation. PC2 explains 13.98% of the total variance with MnSOD, AChE and SH as the parameters that contributed most to the separation. The summary of the results of PCA for all three investigated groups of tissues considering all parameters of oxidative stress (Fig. 1B) indicate that PC1 and PC2 can explain 100% of the total variance. PC1 (69.04%) clearly discriminates BT from CT and MT, while PC2 (33.03%) distinctly discriminates CT from BT and MT.

**Table 2:** Loadings of variables onto the principal components (PC).

	PC1	PC2	PC 3	PC 4	PC 5	PC6	PC7	PC8	PC 9
TotSOD	0.19632*	0.05031	0.00344	0.08984	0.02378	0.01336	0.10524	0.00179	0.00824
MnSOD	0.03295	0.21251*	0.09967	0.04831	0.45251	0.03263	0.00535	0.04840	0.06242
CuZnSOD	0.18793*	0.07656	0.00857	0.08021	0.00770	0.01869	0.11474	0.00435	0.01405
CAT	0.11868	0.00148	0.03229	0.01474	0.00583	0.31247	0.44414	0.00147	0.06886
GSH-Px	0.13793	0.00094	0.11683	0.00361	0.09515	0.00012	0.03514	0.21178	0.37846
GST	0.15190*	0.00291	0.03913	0.14870	0.02425	0.21803	0.00645	0.15858	0.27000
GR	0.15835	0.05164	0.00243	0.00639	0.02184	0.15547	0.02808	0.55501	0.02075
GSH	0.00282	0.05271	0.45076	0.08098	0.31966	0.03820	0.03451	0.00031	0.02001
AChE	0.01175	0.28401*	0.04002	0.24425	0.00236	0.21084	0.20241	0.00412	0.00022
SH	0.00133	0.26688*	0.20680	0.28293	0.04688	0.00016	0.02389	0.01415	0.15694
Eigenvalues	3.80730	1.39806	1.18163	0.99755	0.78241	0.72850	0.54598	0.29637	0.26215
% of each axis	38.07%	13.98%	11.82%	9.98%	7.82%	7.28%	5.46%	2.96%	2.62%

The parameters that contributed most to the separation are marked in bold and with an asterisk.

### Concentrations of trace elements

There were only a few significant changes in the concentrations of investigated trace elements (Table 3). The concentration of Mn was markedly lower in patients with malignant brain tumors compared to both CT (*P* = 0.048) and BT (*P* = 0.041) groups. Similar results were obtained for Cu concentration (CT *vs* MT, *P* = 0.032; BT *vs* MT, *P* = 0.02). Decreased Mn concentration could be associated with decreased MnSOD enzyme activity in patients with malignant brain tumors. Increased concentration of Zn, and significantly reduced Cu concentration in the malignant tumors, could be associated with decreased CuZnSOD enzyme activity in the MT group compared to CT. The concentration of Se was not statistically significantly changed in both BT and MT and agrees with the unchanged activity of Se-dependent GSH-Px activity, which is the main fraction of GSH-Px isoenzymes, even though its concentrations were highest in the group with benign brain tumors. Finally, the malignant brain tumors have reduced concentrations of Mn and Cu compared to both healthy brain tissue and benign brain tumors.

**Table 3:** Concentration of specific trace elements in control brain tissue (CT), benign tissue (BT) and malignant brain tissue (MT) expressed in (µg/g).

µg/g	Control tissue (CT)	Benign tissue (BT)	Malignant tissue (MT)	CT *vs* BT	CT *vs* MT	BT *vs* MT
Mn*	99 (118)	104 (102)	78.3 (66.3)	0.889	0.048*	0.041*
Cu*	1269 (1555)	1293 (2252)	675 (905)	0.603	0.032*	0.020*
Zn*	3204 (3401)	4924 (3082)	3956 (4188)	0.395	0.352	0.557
Se*	110 (170)	174 (124)	142 (121)	0.555	0.660	0.617

The data are expressed as the median (interquartile range). Difference between groups was determined by nonparametric Mann–Whitney U test for independent groups with *P* < 0.05 as the level of significance. Differences between groups are marked as bold and with the asterisk (**CT *vs* BT; CT *vs* MT; BT *vs* MT**).

*According to the World Health Organization Geneva, 1996: Trace elements in human nutrition and health. Trace elements—standards.

### Correlation analysis

Generally, correlation analysis provides us with information about the interconnectedness between investigated parameters. The criteria for correlation strength were previously explained in the paper by Ratner^13^ While our results show many positive and negative correlations, all of them are of medium strength, except for strong correlations between AChE-Mn and AChE-Cu in BT samples, as well as weak correlations between AChE-Se in BT samples and MnSOD-Cu in MT samples (Table 4).

**Table 4:** Calculation results of nonparametric Spearman’s rank correlation coefficients between oxidative stress parameters and selected trace elements in control brain tissue (CT), benign tissue (BT) and malignant tissue (MT).

	CT *ρ*				BT *ρ*				MT *ρ*			
	Mn	Cu	Zn	Se	Mn	Cu	Zn	Se	Mn	Cu	Zn	Se
TotSOD								0.54 (0.015)	0.35 (0.04)	0.47 (0.024)		
MnSOD									0.56 (0.012)	0.29 (0.049)		
CuZnSOD								0.53 (0.017)	0.30 (0.047)	0.46 (0.025)		
CAT					− 0.54 (0.015)					0.37 (0.037)		
GSH-Px					− 0.47 (0.024)	− 0.65 (0.005)	− 0.64 (0.006)					
GST								0.48 (0.023)				
GR	− 0.44 (0.030)		− 0.44 (0.030)				− 0.49 (0.022)		0.31 (0.045)			
AChE				0.51 (0.019)	0.80 (< 0.01)	0.90 (< 0.01)	0.53 (0.017)	0.29 (0.049)				
GSH								0.57 (0.010)				
SH			0.50 (0.02)	0.47 (0.025)			0.51 (0.019)	0.55 (0.014)				

*Exact *P* values are in parentheses.

In the CT group, negative correlations were obtained between GR and Mn (*ρ* = − 0.44) and Zn (*ρ* = − 0.44); positive correlations were observed between AChE and Se (*ρ* = 0.51) and between the SH groups and Zn (*ρ* = 0.50) and Se (*ρ* = 0.47). The P values are given in Table 4.

In patients with benign brain tumors, we obtained positive correlation between TotSOD and CuZnSOD with Se (*ρ* = 0.54 and 0.53, respectively). Negative correlations were obtained between CAT and Mn (*ρ* = − 0.54), as well as between GSH-Px and Mn (*ρ* = − 0.47), and Cu (*ρ* = − 0.65) and Zn (*ρ* = − 0.64). A positive correlation was found between GST and Se (*ρ* = 0.48) and a negative correlation between GR and Zn (*ρ* = − 0.49). Positive correlations were also obtained between GSH, AChE and SH groups versus Se (*ρ* = 0.57, 0.29 and 0.55, respectively). AChE also showed positive correlations with Mn (*ρ* = 0.80), Cu (*ρ* = 0.90), and together with SH groups, with Zn (*ρ* = 0.53 and 0.51, respectively).

In the group with malignant brain tumors, positive correlations were obtained between TotSOD, MnSOD, CuZnSOD and Mn (*ρ* = 0.35, 0.56 and 0.30, respectively) and Cu (*ρ* = 0.47, 0.29 and 0.46, respectively). CAT displayed a positive correlation with Cu (*ρ* = 0.37), and GR with Mn (*ρ* = 0.31). Most correlative relationships were established with Se in the BT group and with Cu in the MT group. The strongest correlative connections were established between AChE and Mn (*ρ* = 0.80) and Cu (*ρ* = 0.90).

## Discussion

While the brain carefully regulates oxygen use, it is susceptible to oxidative stress, and neuronal sensitivity to oxidative stress oscillates. According to Halliwell^14^, the brain’s low endogenous antioxidant defense relative to many other tissues (the liver for example) renders it more susceptible to oxidative stress and disrupted redox homeostasis. Neurons possess a 50-fold lower CAT content compared to hepatocytes, while cytosolic GSH is about 50% lower in neurons compared to other cells, which restricts GSH-Px activity. Cobley et al.^15^ singled out 13 reasons for brain susceptibility to oxidative stress. The most important are its enrichment with unsaturated lipids, glucose concentration, mitochondria, calcium, glutamate, its relatively low antioxidant defense, the presence of (relatively high concentrations of) redox active transition metals, neurotransmitter auto-oxidation and RNA oxidation. Altered brain transmitter balance can also cause a disturbance in antioxidant defense. Intracellular cystine is reduced to cysteine that can be used by glutamate cysteine ligase for de novo glutathione (GSH) synthesis. Inhibition of cystine uptake causes oxidative stress as the result of depletion of intracellular GSH. The high unsaturated lipid content in the brain can also enhance oxidative stress. The phospholipids of cerebral cell membranes are enriched in polyunsaturated fatty acids (PUFAs) which together with the low activity of brain antioxidant enzymes and the high content of pro-oxidant metal ions (Fe^2+^, Cu^2+^, Co^2+^, and Cr^3+^) render the brain vulnerable to lipid peroxidation. The deleterious consequence of lipid and protein oxidation is the induction of pro-inflammatory enzymes that intensify brain inflammation^16^.

Tumorigenesis has been related to both increased levels of free radicals as inductors of severe damage in healthy cells, but also with the reduced response of endogenous enzymes and nonenzymatic antioxidant defenses^17^.

Zalewska-Ziob et al.^18^ noted a significant change in the activity of antioxidant enzymes during the process of cancerogenesis. The results of their study agree with ours, and the same trend was detected in the examined parameters. The authors found that tumor cells always had low MnSOD activity, typically low CuZnSOD activity, and almost always low CAT activity compared with matching normal tissues. The activities of GSH-related enzymes were significantly higher in lung cancer tissues compared with adjacent noncancerous tissues. In our study, GSH-related enzyme activities were higher in malignant tissues, which could be a possible way of tumor cells protecting themselves from increased oxidative stress.

The activities of TotSOD and MnSOD were found to be significantly higher only in non-cancerous tissues of patients with squamous cell carcinoma, while their activities did not change significantly in patients diagnosed with adenocarcinoma^18^.

Patients with liver cancer had 22% lower CAT activity compared to non-cancerous individuals^19^. Also, De Craemer et al.^20^ noticed that CAT activity in the liver was reduced in patients with malignant disease. In breast cancer, higher ROS production and decreased CAT activity indicated oxidative stress^21^. The reduced catalase activity may lead to the accumulation of reactive oxygen metabolites, and probably to the initiation of carcinogenesis. This change in catalase activity suggests a possible link between decreased antioxidants and increased free radical levels, which also confirms the existence of oxidative stress in malignant cancers.

Aggarwal et al.^22^ showed that malignant tumors exhibited relative decreases in antioxidant enzyme levels as compared to benign tumors, which was not always the case in our study. Comparison of histopathological sections of brain tumors also suggested an inverse relationship between antioxidant levels and grades of malignancy^22^.

Nomani et al.^23^ found that plasma GST activity was significantly higher in colorectal cancer patients than in healthy individuals and suggested that GST measurement could serve as a biomarker in colorectal cancer. According to Prabhu and Bhat^24^, alterations in serum total GST levels could play a role in cancer progression. GST activity in the MT group in our investigations was markedly higher with respect to both CT and BT.

Cholinesterases have an essential role in the transmission of nerve impulses and represent indicators of neurotoxicity^25^. It has been confirmed that heavy metal ions can alter ChE activities, causing various post-translational modifications of ChE proteins^26^. In view of the lower concentrations of Mn and Cu ions in the MT group and the strong correlations between AChE and Mn (0.80) and Cu (0.90), we hypothesized a connection between lower AChE activity and the concentrations of Mn and Cu in the MT group.

One of the most important non-enzymatic antioxidants is GSH. A decrease in GSH level is usually accompanied by a simultaneous increase in oxidized glutathione (GSSG), as was reported in Alzheimer’s and Parkinson’s diseases, multiple sclerosis, amyotrophic lateral sclerosis, and Huntington’s disease^27^. GSH participates in the regeneration of other antioxidants, it regulates gene expression, maintains SH groups in the reduced state and affects cell proliferation and differentiation and neuronal apoptosis^27^. In our study, we observed significantly increased concentrations of the SH group in BT patients, which is associated with an increase of GSH levels in the same group of patients (BT) when compared to CT and MT. Measurement of total thiols is a particularly useful parameter because it provides reliable data on their consumption and is closely related to the activities of enzymes that use thiols to perform their functions. Thus, we can indirectly conclude about the state of the GSH-dependent antioxidant status.

Mn is an integral part of the active center of the MnSOD enzyme. In our experiments, we obtained significantly lower concentrations of Mn in both BT and MT groups. Decreased Mn concentration could be associated with decreased MnSOD enzyme activity especially in patients with malignant brain tumors, which confirms to us the presence of significant positive correlations between MnSOD and Mn.

Cu is necessary for the process of neural myelination. It is also involved in the metabolism of neurotransmitters and in the regulation of other metals in the body, primarily Zn^28^. Zn is found in high amounts in brain tissue, where it assumes catalytic, structural, and regulatory roles in cellular metabolism^29^. One of the most abundant essential trace metals in the brain is Zn which is involved in several functions^30^. Zinc is a structural and catalytic component of CuZnSOD and provides stability to transcription factors, it assumes neuroprotective properties, and it is also involved in the defense against oxidative stress^31,32^. In the brain, Zn has key roles in synaptic plasticity, in the regulation of neurogenesis, neuronal migration and differentiation and in the modulation of neurotransmission^33,34^, thereby mediating healthy cognitive development and brain functioning^35^. It is possible that the cancerous cell consumes the Zn present in the circulation for tumor growth and maintenance of membrane integrity. This might be a possible reason for the observed depletion of Zn in cancer patients^36^. Zinc interacts with other trace elements and is an antagonist to copper. Copper in the brain is an essential a co-factor for CuZnSOD mediating the oxidative stress response and neurotransmitter biosynthesis. For example, in enzyme CuZnSOD, Cu provides the catalytic activity for the antioxidant enzyme CuZn-SOD while Zn plays a critical structural role^37^.

In the study of Arslan et al.^38^, Cu levels in 22 patients with malignant gliomas were studied and they decreased in patients with malignant glioma in comparison to healthy subjects. Our results support these findings, considering that the obtained Cu values were significantly lower in the MT than in the CT and BT groups.

Selenium (Se) is an important element that takes part in different brain functions, such as motor performance, coordination, memory, and cognition, acting as a neurotransmitter and has protective properties against oxidative damage^39^. Therefore, Se deficiency could cause irreversible changes in neuronal cells. Se is also a component of the most dominant Se-dependent GSH-Px isoenzyme of the GSH-Px family. Our results showed that there was no change in the concentration of Se in either benign or malignant tumor tissue, which is in accordance with the unchanged activity of the GSH-Px enzyme.

The antioxidant system completely changes its functioning in malignant tissue, which can be observed as changes in the correlation of system parameters. Among the many obtained correlations between antioxidant enzymes and trace elements, most were linked to Se in the BT group and Cu in the MT group. These trace elements mainly correlated with SOD isoenzymes, but the strongest correlative interrelationships were obtained between AChE and Mn and Cu, which is not surprising since all parameters were significantly decreased in malignant tissue.

As stated, PCA was performed to determine which component of the antioxidant defense contributed most to the changes observed in the investigated tissues. PCA pointed to two important conclusions: that there were clear differences between the tissues, between BT towards CT and MT (according to PC1), and that there were differences between the tissues, namely CT towards BT and MT (according to PC2). The parameters that contributed most were TotSOD, CuZnSOD and GST (PC1), and MnSOD, AChE and SH (PC2). Our previous investigations^40^ also revealed a clear separation of antioxidant defense parameters in the blood of control individuals and patients with brain tumors and hydrocephalus, with a clear separation between patients with brain tumors and hydrocephalus.

Patients with benign and malignant brain tumors had increased oxidative stress, and those with malignant brain tumors exhibited an impaired equilibrium of trace elements (most important are TotSOD, MnSOD and GST activities and SH concentration). Correlations between antioxidant parameters and trace elements revealed good synergistic action of antioxidant mechanisms and a cooperative response to the pro-oxidation process. Bearing in mind that lowered antioxidant enzyme activities are indicative of oxidative stress, the observed alterations point to the importance of oxidative stress in disrupting brain homeostasis and its role in the process of pathogenesis of both benign and malignant brain tumors. The obtained results indicate that individuals with brain tumors displayed specific changes in antioxidant status; thus, in patients with benign brain tumors, decreased TotSOD and MnSOD activities and increased SH group concentrations were observed, while patients with malignant tumors were characterized by increased GST activity and decreased AChE activity. It should be emphasized that the malignant brain tumors have reduced concentrations of Mn and Cu compared to both healthy brain tissue and benign brain tumors.

Examination and comparative analysis of antioxidant system parameters in malignant and benign tumor tissues, together with normal tissues, could be particularly important for understanding the role of ROS as well as AOS in the pathophysiology of brain cancer. Also, the detection of sensitive and specific/selective biomarkers for systemic oxidative damage could be essential for understanding the role of oxidative stress in human diseases, especially in brain cancer.

## Materials and methods

### Patients and samples

Subjects in all groups had average weights and heights, while the body mass index (BMI) was within normal values (between 18.5 and 23 in every group).

To avoid additional oxidative pressure, smokers, patients with other malignant disease and/or liver, kidney, and lung diseases, as well as those patients with autoimmune conditions were excluded. Individuals’ medical histories confirmed that the patients did not have any therapy nor take any supplements and were on a normal diet.

The study covered three important types of clinical samples: control tissue (CT), benign tumors (BT) and malignant tumors (MT). Control/healthy and tumor brain tissue samples were obtained from patients undergoing craniotomy for the removal of an intracranial tumor, immediately after surgical intervention at the Department of Neurosurgery Clinical Center of Serbia. Tumor tissues of 60 patients with benign brain tumor (f/m ratio = 34/26; mean age for female: 38 ± 4 and male: 40 ± 5) and 60 patients with malignant brain tumor (f/m ratio = 25/35; mean age for female: 41 ± 5 and male: 37 ± 4) were analyzed. CT without apparent tumor infiltration was taken from each patient (n = 60) with benign tumors. These samples were collected from patients at the greatest possible distance from the BT, which were well demarked from the rest of the tissue. Histopathological analysis of CT did not reveal any pathological entity. Preoperative diagnosis was carried out using imaging techniques such as X-ray computed tomography and/or magnetic resonance imaging. A definitive diagnosis of BT and MT was confirmed by two independent neuropathologists after postoperative pathohistological analysis of brain tissues. Tissue samples were collected over two years (2017–2019). All collected specimens were treated in the same way to perform an adequate comparative analysis between groups.

All patients voluntarily participated in this research and written informed consent from all study participants and / or their legal guardians was obtained. The study was conducted in full compliance with the principles of the World Medical Association Declaration of Helsinki^41^ “Ethical Principles for Medical Research Involving Human Subjects” (Helsinki, 1964, as amended during 1975–2013) and current legislative and governmental regulations of the Republic of Serbia. Approval for this study was obtained by the Ethics Committee of the Clinical Center of Serbia Belgrade (EC approval from 19.10.2017, number 442/3).

### Preparation of tissues

The sample of brain tissue was taken during open surgery from the periphery of the lesion, not less than 1 cm^3^, which is about 1 g of tissue, and blood and visible blood vessels were removed. Precise adjustment to 1 g of tissue was performed in the laboratory by sample reduction to the required weight. After excision, tissues were washed in 0.9% NaCl and immediately frozen in liquid nitrogen (− 196 °C) and stored at − 80 °C until analysis for no longer than one month. The tissues were minced and homogenized on ice in 5 volumes of 25 mmol/L sucrose containing 10 mmol/LTris-HCl, pH 7.5, supplemented with 1 × phosphatase-inhibitor Mix I and 1 × protease-inhibitor Mix G at 4°C^42^, using an IKA-Werk Ultra-Turrax homogenizer (Janke and Kunkel, Staufen, Germany)^43^. The homogenates were sonicated for 30 s at 10 kHz on ice to release enzymes^44^, followed by centrifugation in a Beckman ultracentrifuge at 100,000 × *g* for 90 min at 4°C^45,46^. Each sample was measured in triplicate and the mean values of three measurements were used for further calculation.

### Antioxidant enzyme assays

The activity of SOD was assayed by the epinephrine method^47^. One unit of SOD activity was defined as the amount of protein causing 50% inhibition of the autoxidation of adrenaline at 26 °C and was expressed as specific activity (U/mg protein). For the determination of MnSOD activity, the assay was performed after pre-incubation with 8 mmol/L KCN. CuZnSOD activity was calculated as the difference between TotSOD and MnSOD activities. CAT activity was evaluated by the rate of hydrogen peroxide (H_2_O_2_) decomposition^48^ and expressed as µmol H_2_O_2_/min/mg protein. The activity of GSH-Px was determined by following the oxidation of nicotinamide adenine dinucleotide phosphate (NADPH), which served as a substrate, with t-butyl hydroperoxide^49^, and expressed in nmol NADPH/min/mg protein. The activity of GST toward 1-chloro-2,4-dinitrobenzene (CDNB) was determined by the method of Habig et al.^50^ and expressed as nmol GSH/min/mg protein. The method is based on the reaction of CDNB with the SH group of GSH, which is catalyzed by GST contained in the samples. The activity of GR was measured using the method of Glatzle et al.^51^, based on the capability of GR to catalyze the reduction of oxidized glutathione (GSSG) to reduced glutathione (GSH) using NADPH as a substrate in phosphate buffer (pH 7.4). GR activity was expressed as nmol NADPH/min/mg protein. Protein concentrations in the supernatants were determined according to the method of Lowry et al.^52^ using bovine serum albumin as standard and expressed in mg/g wet mass. The activities of the antioxidant enzymes were measured simultaneously in triplicate for each sample using a Shimadzu UV-1800 spectrophotometer with a temperature-controlled cuvette holder at 37 °C. All chemicals were obtained from Sigma-Aldrich (St Louis, MO, USA).

### Nonenzymatic antioxidant profile

The concentration of total GSH was determined by the method of Griffith^53^ and expressed as nmol/g of tissue. The concentrations of SH groups were determined using DTNB according to the Ellman^54^ method and expressed in µmol/g wet mass. The parameter of neurotoxicity, AChE activity, was determined according to Ellman et al.^55^. The assay consisted of measuring the reaction of thiol with of 5,5′- dithiobis-(2-nitrobenzoic acid) (DTNB). The yellow anion of 5-thio-2-nitrobenzoic acid formed in the reaction was detected spectrophotometrically at 412 nm.

### Analysis of trace elements in tissues

About 0.5 g of each sample was transferred into a microwave vessel and the exact mass was measured. All samples were decomposed at 180ºC in a mixture of nitric acid (65%) and hydrogen peroxide (30%) at a 4:1 ratio (v/v). Digestion was carried out under the following microwave program: 10 min warm-up to 180ºC and heating for additional 20 min. After cooling to room temperature, decomposed samples were transferred into volumetric flasks and diluted with ultrapure water to 25 mL. All elements were quantified by inductively coupled plasma-mass spectrometry, ICP-MS (iCAP Q_*c*_, Thermo Scientific, UK) in the optimized mode of action and by applying the internal standardization. Good linearity for each element (R > 0.99) was obtained in the range from 1 to 250 µg/L. The accuracy of the ICP-MS was controlled by SRM (Seronorm™ Trace Elements Whole Blood Level-1). The obtained recovery values for elements in the SRM was in the range from 94.2 to 105%.

### Statistical analysis

The data were presented as the mean value ± standard error (SE). The normality of the data was checked by the Kolmogorov–Smirnov test. Since the test of normality did not show a normal distribution of samples, the data were log transformed and then further statistically analyzed. One-way analysis of variance (ANOVA) was performed to determine all interactive effects between the investigated tissues. When an interactive effect was observed, Fisher’s LSD (least significant difference) post-hoc test was used to obtain significant differences among the means for equal N (sample number). Principal component analysis (PCA) was implemented to statistically determine the differences between the investigated groups based on all investigated antioxidant defense biomarkers and to examine variables that significantly contributed to differences in the investigated parameters. The difference between groups in trace-element concentrations was determined by the nonparametric Mann–Whitney U test for independent groups. Nonparametric Spearman’s rank correlation analysis was also conducted. A minimum significance level of *P* < 0.05 was accepted for all cases. All data were processed using the statistical package Statistica 10.0.

## Supplementary Information

Supplementary Information.
