# Supplementary material for: Differences between antioxidant defense parameters and specific trace element concentrations in healthy, benign, and malignant brain tissues
Source: Sci Rep. 2021 Jul 20;11:14766. doi: 10.1038/s41598-021-94302-5 (PMC8292338; doi:10.1038/s41598-021-94302-5)
Supplement: Supplementary file 1 — Supplementary Information. [file 41598_2021_94302_MOESM1_ESM.pdf]

Supplementary Material

Differences between antioxidant defense parameters and specific trace element concentrations in healthy, benign, and malignant brain tissues

Slavica Borković-Mitić[1*], Aleksandar Stojsavljević[2, 3], Ljiljana Vujotić[4, 5], Siniša Matić[5], Bojan Mitić[6], Dragan Manojlović[2, 7] and Slađan Pavlović[1]

Table S1. Results of one-way ANOVA of the comparison between different investigated tissues. **SS** – Sum of Squares-Variance; F – F variable, the ratio of two independent chi-squared variables divided by their respective degrees of freedom; MS – Mean Square; df – degrees of freedom.

Variable	*SS*	*F*	*MS*	*df*
TotSOD	6.23303	2.783904	0.067022	177
MnSOD	2.04991	3.744776	0.022042	177
CuZnSOD	9.37584	0.980071	0.100815	177
CAT	8.03281	0.945543	0.086374	177
GSH-Px	11.01009	2.251251	0.118388	177
GR	11.11263	2.199384	0.119491	177
GST	7.38116	0.984841	0.079367	177
GSH	10.12841	0.215556	0.108908	177
AChE	8.13960	1.443830	0.087523	177
SH	12.05807	3.068739	0.129657	177

TotSOD – total superoxide dismutase; MnSOD – manganese containing superoxide dismutase; CuZnSOD – Cu/Zn-containing superoxide dismutase; CAT – catalase; GSH-Px – glutathione peroxidase; GST – glutathione S-transferase; GR – glutathione reductase; AChE – acetylcholine esterase; GSH – glutathione; SH – sulfhydryl groups.
